# Aging is associated with a prefrontal lateral-medial shift during picture-induced negative affect

**DOI:** 10.1093/scan/nsx144

**Published:** 2018-01-08

**Authors:** Carien M van Reekum, Stacey M Schaefer, Regina C Lapate, Catherine J Norris, Patricia A Tun, Margie E Lachman, Carol A Ryff, Richard J Davidson

**Affiliations:** 1 Centre for Integrative Neuroscience and Neurodynamics, School of Psychology and Clinical Language Sciences, University of Reading, Reading, RG6 6AL, UK; 2 Waisman Brain Imaging Core & Center for Healthy Minds, University of Wisconsin-Madison, Madison, WI 53706, USA; 3 Department of Psychology, Brandeis University, Waltham, MA 02453, USA,; 4 Institute on Aging, University of Wisconsin-Madison, Madison, WI 53706, USA

**Keywords:** aging, emotion, VMPFC, VLPFC, executive function

## Abstract

The capacity to adaptively respond to negative emotion is in part dependent upon lateral areas of the prefrontal cortex (PFC). Lateral PFC areas are particularly susceptible to age-related atrophy, which affects executive function (EF). We used structural and functional magnetic resonance imaging (MRI) to test the hypothesis that older age is associated with greater medial PFC engagement during processing of negative information, and that this engagement is dependent upon the integrity of grey matter structure in lateral PFC as well as EF. Participants (*n =* 64, 38–79 years) viewed negative and neutral scenes while in the scanner, and completed cognitive tests as part of a larger study. Grey matter probability (GMP) was computed to index grey matter integrity. FMRI data demonstrated less activity in the left ventrolateral PFC (VLPFC) and greater ventromedial PFC (VMPFC) activity with increasing age during negative-picture viewing. Age did not correlate with amygdala responding. GMP in VLPFC and EF were negatively associated with VMPFC activity. We conclude that this change from lateral to medial PFC engagement in response to picture-induced negative affect reflects decreased reliance on executive function-related processes, possibly associated with reduced grey matter in lateral PFC, with advancing age to maintain emotional functioning.

## Introduction

As we age, we experience a loss in brain matter with an associated decline in cognitive function (Ziegler *et al.*, 2010). Age-related brain atrophy starts during young adulthood, with a steeper decline after age 50 years (Fotenos *et al.*, 2008). This neural loss is not equally distributed across the brain: grey matter in lateral prefrontal cortical (PFC) areas is most vulnerable to the effects of aging, while grey matter in ventromedial areas of the PFC (VMPFC) and the amygdala remains relatively preserved across age (Raz *et al.*, 2004; Lemaitre *et al.*, 2012). These lateral PFC areas support executive function, where complexity of representations and control functions are proposed to follow a rostro-caudal gradient of PFC (see Badre, 2008). Similar lateral PFC areas also form key components of corticolimbic networks supporting emotional processes, as highlighted in human neuroimaging studies (for relevant meta-analyses, see Buhle *et al.*, 2014; Kober *et al.*, 2008). Areas in VMPFC underlie the flexible control of (negative) affect (Schiller and Delgado, 2010), and evidence suggests that areas in VMPFC mediate the inverse functional association between lateral and dorsomedial areas of PFC and the amygdala measured during voluntary downregulation of negative emotion (Urry *et al.*, 2006; Johnstone *et al.*, 2007). Collectively, these findings underscore the importance of lateral and medial PFC engagement in emotional processing.

Because the lateral PFC areas demonstrating age-related atrophy with an associated decline in ‘Executive Function’ (EF) overlap with PFC areas involved in emotional processing, a logical hypothesis would then be that the capacity to adaptively respond to emotional information declines with age. Recent brain imaging evidence, however, suggests that, relative to their younger counterparts, older-aged individuals engage different cortical networks, involving dorsal areas in medial PFC when processing emotional facial expressions (e.g. Williams *et al.*, 2006), or VMPFC specifically when processing complex negative images (St. Jacques *et al.*, 2010; Dolcos *et al.*, 2014). These studies did not find age-related changes in the activity of the amygdala, a key region for the acquisition and expression of emotion (Davis and Whalen, 2001), suggesting that aging impacts activity in PFC systems computing negative affect, without necessarily altering the emotional response (see also van Reekum *et al.*, 2011). Thus, engaging medial rather than lateral PFC areas with advancing age may indicate a ‘compensatory change’ (cf. Reuter-Lorenz and Park, 2010) in processing of emotion-laden information, preserving emotional reactivity.

Yet, it is unclear why aging would be associated with such compensatory increased medial PFC activity. One potential reason is that age-related gray matter loss in lateral areas of the PFC, and associated loss in EF, necessitates such a change in processing. Mirroring suggestions of age-related compensatory neural mechanisms in a variety of cognitive functions (Reuter-Lorenz and Park, 2010; Kalpouzos *et al.*, 2012; Meunier *et al.*, 2014), and optimization of emotion regulatory strategies to compensate for a loss of (cognitive) resources (cf. Urry and Gross, 2010), we hypothesize (1) lower lateral PFC engagement when processing emotionally negative information, and (2) greater medial PFC engagement to compensate for brain matter loss and associated EF in normal aging.

Collected in association with the Midlife in the US (MIDUS) study, we used structural and functional MRI in an adult population aged 38–79 years to test our hypothesis of an age-related shift in PFC function when processing negative information. We also assessed the extent to which such a shift was dependent upon grey matter probability, as an indicator of grey matter integrity in lateral PFC, and EF. Participants (*n =* 64, 38–79 years) viewed negative and neutral scenes from the International Affective Picture System (IAPS, Lang *et al.*, 2008) while in the MRI scanner. Participants also completed a battery of cognitive tests, adapted for administration by telephone (Lachman *et al.*, 2014). We predicted that age would be associated with lower activity in lateral PFC and increased recruitment of medial PFC regions. If grey matter loss in lateral PFC regions and associated loss of EF indeed drive the need for functional compensation, then the grey matter probability and EF should be associated with a functional increase in medial areas of PFC. Because overlapping regions in the lateral PFC subserve both emotion and EF, we furthermore predicted that age-related functional differences in PFC would be stronger for those with relatively lower EF. Finally, we assessed whether age and any age-related differences in PFC function supporting emotional processing was associated with amygdala function.

## Materials and methods

### Participants

Seventy-two participants of the national Midlife in the US (MIDUS II, see http://midus.wisc.edu/) study who lived in the Midwest region of the United States agreed to participate in our MRI experiment, performed at the Waisman Brain Imaging Core, University of Wisconsin-Madison. Because eight participants were excluded due to excessive motion and lack of compliance, 64 participants’ data are included in the analyses [mean age = 58.20 years (s.d. = 11.42), range = 38–79 years, 62 right-handed, 45 females]. Forty-two participants identified themselves as ‘Caucasian’, 20 identified themselves as ‘Black or African-American’, and 2 identified themselves as ‘Native American, Alaska National, Aleutian Islander or Eskimo.’

### Executive function

Cognitive function was assessed over the telephone, using a battery of tests adapted for telephone administering [Brief Test of Adult Cognition by Telephone (BTACT), Lachman *et al.*, 2014]. The BTACT includes assessments of working memory span and executive function, as well as episodic verbal memory, reasoning, and speed of processing. Based on factor analysis, an ‘Executive Function’ (EF) composite score across five BTACT components was created across the entire MIDUS sample for which BTACT data were available (*n =* 4512). The scores for each test detailed below were z-scored prior to averaging the scores into an EF composite score. Following a brief hearing test, scores on the BTACT tasks included in the EF composite score were obtained as follows:

#### Backward digit span

Participants were asked to repeat an increasingly longer series of digits, ranging from 2 to 8. Two sets were presented to the listeners at a rate of 1 s per digit, starting with the shortest series. The score was set to the largest set size correctly reproduced in reverse order across the two sets.

#### Verbal fluency

Participants were given 1 min to provide as many unique exemplars of the category ‘animals’ as possible. The score was the number of unique words produced.

#### Number series completion

Participants were read a sequence of numbers and asked to provide a number that would best continue the sequence. The experimenter read each number aloud, and the participant indicated by saying ‘OK’ that s/he was ready for the next number, until the experimenter prompted a response. Five sequences were presented of varying difficulty, and the score pertained to the number of series correctly completed.

#### Backward counting

Speed in counting backwards from 100 in steps of 1 in 30 s was recorded as the total number of correct responses provided.

#### Stop and Go Switch Task (SGST)

For baseline trials, participants were asked to respond as quickly as possible ‘go’ to the word ‘green’ provided and ‘stop’ to the word ‘red’. For the reverse trials, participants responded by saying ‘stop’ to ‘green’ and ‘go’ to ‘red’. The task included a block of 20 baseline and a block of 20 reverse trials, followed by a mixed block of 32 trials, where the participant was given eight cues to switch following 4–6 items of each trial type. The median latency (RT) within the mixed block was calculated across the correct trials following the switch cue and the remaining correct trials, and reverse scored for inclusion in the EF composite score.

### Imaging task

Sixty negative, 60 neutral, and 60 positive images^1^ were selected from the International Affective Picture System on the basis of the normative ratings provided with this set (IAPS; Lang *et al.*, 2008), and carefully matched for luminosity, picture complexity (using the jpeg size as an index of complexity) and social content. Stimulus presentation was accomplished using E-Prime software (Psychology Software Tools, Inc., Pittsburgh, PA), while visual stimulation was delivered via a fiber-optic goggle system (Avotec, Inc., Stuart, FL). A white fixation cross was displayed in the center of a black screen for 1 s, followed by a picture presented for 4 s. An intertrial interval, selected from an exponential distribution and varied from 5.5 to 17.6 s with an average duration = 8.89 s, consisted of a black screen with a white fixation cross. Two randomized orders of conditions were randomly allocated to participants, and pictures were randomly allocated to each of the conditions for each participant.

A day prior to the scanning session, participants underwent a separate simulation session to acquaint them with the scanning environment and to train on performing the tasks. Participants received detailed instructions on what to expect during the actual scanning session and were instructed that ‘It is important that you watch each picture the entire time it is on the screen, without closing your eyes or looking away. Also, even after the picture disappears from the screen, please keep your eyes open and keep looking at the white cross in the middle of the screen (the “fixation” cross).’ In addition, to ensure alertness during the task, participants were instructed to ‘press one of three buttons to indicate whether s/he thought the picture was positive (pleasant), neutral, or negative (unpleasant). They were told “From time to time, you will see a face following the color picture. You don’t need to respond to the face. Instead, focus on the task of responding to the color pictures. There is no right or wrong response in this task. What is important to us is what you think of the pictures. Also, rely on your first impression; don’t think too much or too long about whether you find the picture positive, negative or neutral”’. We recorded the valence choice and judgment time (cf. van Reekum *et al.*, 2007). After a short practice, participants were positioned inside the bore of an inactive MRI scanner shell complete with bed, head coil, response box, and stimuli presentation goggles, and completed an additional 18 practice trials of the picture task. The pictures used for this practice were representative, but different from the pictures used in the actual scanning task. The experimental scanning session occurred on the morning following the simulation session.

For each valence, on 40 of the 60 trials, a neutral male face was presented either 1 s or 3 s following offset of the IAPS picture. Because our focus is on the neural response to the negative images, we solely examined the BOLD response to the 20 negative and 20 neutral image conditions without face presentations.

### Image acquisition

Images were acquired on a General Electric (Fairfield, CT) SIGNA 3.0 tesla high-speed MRI scanner at the Waisman Brain Imaging Core with a standard clinical whole-head transmit-receive quadrature head coil. Functional images were acquired using a T2*-weighted gradient-echo, echo planar imaging (EPI) pulse sequence [30 sagittal slices, 4 mm thickness with 1 mm gap; 3.75 × 3.75 mm in-plane (64 × 64 voxels); FOV = 240; repetition time (TR)/echo time (TE)/Flip, 2000 ms/30 ms/60°; 262 whole-brain volumes per run]. Functional images were collected in five runs of approximately 8 min each. Immediately following the functional scans, a high-resolution T1-weighted anatomical image was acquired (T1-weighted inversion recovery fast gradient echo; 256 × 256 in-plane resolution; 240 mm FOV; 124 × 1.1 mm axial slices).

### Image analysis

The first three volumes of each run allowed for stabilization of the magnetic field and were discarded during reconstruction. FMRI data were slice-time and motion corrected using AFNI (Cox, 1996) and fieldmap corrected using in-house software. EPI data were normalized to the 2 mm MNI152 template using FSL’s linear normalization algorithm FLIRT (Jenkinson *et al.*, 2002) and smoothed (5-mm full-width at half-maximum). Individual subject GLMs included regressors to model each of the nine trial types (3 valenced images [positive, negative, neutral] × 3 face probe conditions [no probe, face probe after 1 s and 3 s]), as well as six motion estimate covariates. Data were modeled using the canonical double gamma HRF provided by FSL’s software (Smith *et al.*, 2004). Using FSL’s 3-level GLM approach, a separate GLM was performed for each run of EPI data. A fixed effects GLM was performed on all five runs for each subject, the results of which were used in the subsequent group analyses. At the group level, a multiple regression analysis examined the relationship between brain activity to negative information (negative—neutral contrast maps) and age. The search space was restricted to the prefrontal cortex, defined by merging the prefrontal cortical regions from the Harvard-Oxford probabilistic cortical structural atlas thresholded at 25% (cingulates, medial and lateral OFC and PFC, operculum, and frontal pole) into one mask. Clusters of brain activity within this search space were identified using Monte Carlo simulations (AFNI’s AlphaSim procedure) to achieve a corrected cluster threshold of *P < *0.05. To examine amygdala activity to the negative images as well as any age-differences in activity, we extracted the parameter estimates across all voxels in two (left and right) amygdala regions of interest (ROIs), defined using the Harvard-Oxford probabilistic subcortical structural atlas thresholded at 50% probability.

To quantify grey matter probability (GMP), we used FSL’s FAST algorithm (Zhang *et al.*, 2001) to segment the high resolution T1-weighted image in grey, white matter, and CSF. We then normalized and blurred the grey matter probability maps to the same extent as the functional data of interest. Clusterwise grey matter probability was extracted using the statistical ROIs from the regression analysis.

Follow-up analyses testing associations between structural and functional data, extracted based on the a-priori and statistical ROIs, were performed using Pearson correlations. The alpha level was set to *P* < 0.05, one-tailed for the predicted associations (GMP with BOLD and age, and executive function with BOLD and age), and *P* < 0.05, two-tailed for all other analyses.

### Picture ratings

After the scan and if time permitted, participants were asked to rate the pictures seen during the task using a computerized version of the evaluative space grid, above which the picture was displayed. For a full description of the grid, the reader is referred to Larsen and colleagues (Larsen *et al.*, 2009). Briefly, the evaluative space provides the simultaneous measurement of both positive and negative feelings to pictures by positioning in a 2-dimensional 5×5 grid space. The grid has been validated against unipolar and dichotomous-then-unipolar ratings (Larsen *et al.*, 2009). For the purposes of reporting the data against the normative data provided with the IAPS, the grid data were recoded into 9-point valence ratings similar to the 9-point valence scales used in IAPS, where 1 indicates very negative and 5 indicates neutral (and 9 indicates very positive).

## Results

### Amygdala ROIs

Both left and right amygdala activity were higher in response to negative (*M* = 0.21, *SE* = 0.020) than to neutral (*M* = 0.16, *SE* = 0.018) images, Valence, *F*(1, 63) = 11.20, *P* = 0.001. Hemisphere did not interact with Valence, *F* < 1. Age was uncorrelated with amygdala activity, both in the left (*r* < 0.1) and right (*r* < 0.1) amygdala.

### Age and picture affect judgments

Participants provided a categorical judgment of each picture whilst in the scanner. Data of three participants were lost due to problems with the button box, leaving *n* = 61. Although the proportion of negative pictures judged as negative (*M* = 0.78, s.d. = 0.17) was higher than the proportion of neutral pictures judged as neutral (*M* = 0.62, s.d. = 0.22) overall, this effect was not significant, *F* < 1, nor was there a significant interaction with age, *F*(1, 59) = 2.45, *P* = 0.12. We then assessed whether the speed with which images were evaluated as negative or neutral differed as a function of age. The time taken to judge the pictures was not significantly different for negative (*M* = 1870 ms, s.d. = 334) or neutral (*M* = 2130 ms, s.d. = 463) pictures, *F* < 1, nor were these judgment times modulated by age, *F*(1, 59) = 1.13, *P* = 0.29.

In addition, 56 participants completed the picture ratings after the scan. Whilst negative pictures were rated as more negative (*M* = 2.53, s.d. = 0.62) than the neutral pictures [*M* = 5.54, s.d. = 0.49, *F*(1, 54) = 27.30, *P* < 0.001], age again did not interact with ratings of picture valence, *F* < 1.

### Age-related changes in PFC structure correlates with function

**Fig. 1.**
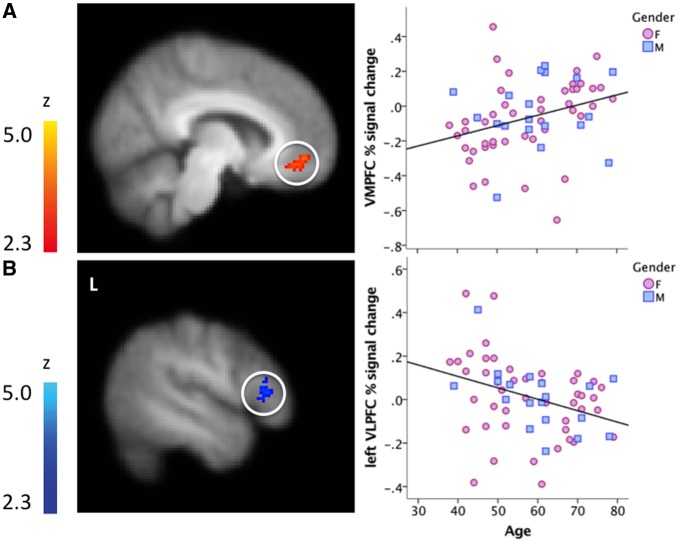
Age predicted BOLD response differences to negative images (vs neutral) in (A) (top) a cluster spanning left and right ventromedial PFC (BA 32/10, volume = 1184 mm^3^, max Z of 3.23 at 6, 48, −8 in MNI space) and in B) (bottom) a cluster in left ventrolateral PFC (BA44/45, pars triangularis of the inferior frontal gyrus extending into pars opercularis, volume = 1256 mm^3^, max Z of 3.24 at 58, 24, 2 in MNI). L = Left. The scatterplots in (A) and (B) depict the relationship between the BOLD response and age, corrected for multiple comparisons, to illustrate that these associations are not carried by outliers. Gender is descriptively depicted by different markers (pink for female, blue for male).

We predicted decreased lateral PFC engagement and increased medial PFC activity in response to negative emotion-eliciting pictures with advancing age. Using a voxel-wise regression predicting age-related changes in BOLD responses to negative pictures (relative to neutral), we found two clusters that survived correction for multiple comparisons: one in the VMPFC (BA32/10, bilateral) and one in left VLPFC (BA45, pars triangularis of the inferior frontal gyrus, extending into BA44, pars opercularis). In line with our predictions, activity in VMPFC was increased and left VLPFC activity was decreased with advancing age (see Figure 1A and B).

**Fig. 2.**
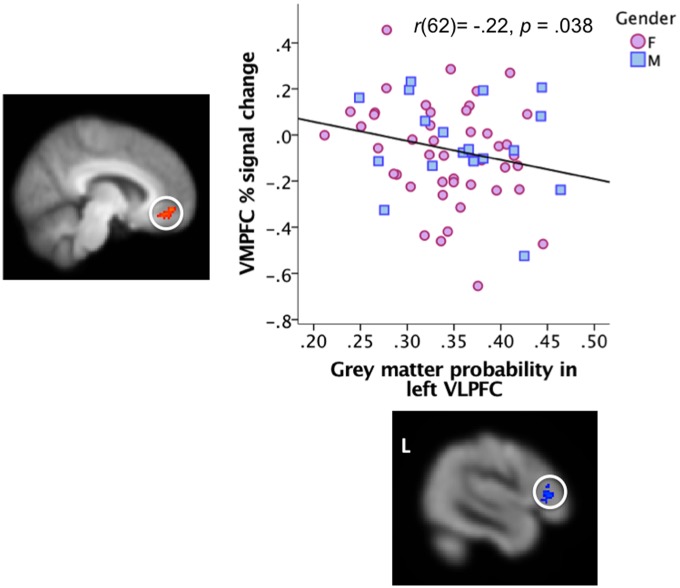
Grey matter probability in the left VLPFC cluster identified in the regression analysis of the functional MRI data correlates negatively with VMPFC BOLD response differences to negative vs neutral images. The scatterplot depicts the relationship, and the images depict the clusters. Gender is descriptively depicted by different markers (pink for female, blue for male). See Figure 1 caption for cluster coordinates and details in MNI space.

We then examined whether gray matter probability (GMP) in these regions may explain this pattern, by correlating (Pearson’s *r*) the average GMP in these statistical ROIs with activity in each of these areas. In line with findings by Raz *et al.* (2004) and Lemaitre *et al.*, (2012), age negatively correlated with GMP in left VLPFC, *r*(62) = −0.43, *P* < 0.001, but not with GMP in VMPFC, *r*(62) = 0.10, *n.s.* Furthermore, activity in VMPFC was negatively correlated with GMP in left VLPFC, *r*(62) = −0.22, *P* = 0.038 (see Figure 2). However, note that GMP in left VLPFC did not mediate the age-VMPFC relationship, Sobel test *t* < 1, indicating that age independently contributed to both GMP changes in VLPFC and to VMPFC activity during negative picture viewing. Suggesting that an age-related reduction in GMP affects the BOLD response, the average BOLD response in left VLPFC was positively correlated with GMP in this ROI, *r*(62) = 0.30, *P* = 0.008. This effect was specific to the left VLPFC area; GMP and BOLD response in VMPFC were not significantly correlated, *r*(62) = 0.12, *n.s.*^2^

### The role of executive function 

Executive function (EF) data were available for 61 of our 64 participants. In line with prior work, EF correlated positively with GMP in left VLPFC, *r*(59)= 0.23, *P* = 0.036. Suggesting a contribution of EF to increased engagement of VMPFC, we found that EF was negatively correlated with VMPFC activity, *r*(59) = −0.21, *P* = 0.047. Note that EF was negatively, but not significantly, correlated with age in our sample, *r*(59) = −0.17, *P* =0.092. EF was not correlated with left VLPFC function, *r*(59) = 0.10, *P* = 0.22.

### Are age-related differences in PFC function associated with amygdala function?

As reported above, age was not correlated with amygdala function in negative emotion processing. Activity in the left VLPFC cluster was positively correlated with left amygdala activity, *r*(62) = 0.22, *P* = 0.043, and with right amygdala, albeit not significantly, *r*(62) = 0.17, *P* = 0.093. These correlations held when controlling for age [VLPFC with left amygdala, *r*(61) = 0.23, *P* = 0.033, and with right amygdala *r*(61) = 0.19, *P* = 0.066]. VMPFC activity was not correlated with left (*r* < 0.1) or right (*r* < 0.1) amygdala activity.

## Discussion

Our findings demonstrate that a shift from left ventrolateral (VLPFC) to ventromedial PFC (VMPFC) engagement characterizes negative affect processing with advancing age, while aging does not affect amygdala function in a picture-based emotion-processing task. Although a number of studies have examined age-related compensation in cognitive brain function, objective assessments of the contribution of age-related structural brain change to those functional changes have only recently been considered (e.g. Teipel *et al.*, 2007; Kalpouzos *et al.*, 2012; Meunier *et al.*, 2014). Using grey matter probability, we established that higher grey matter probability in left VLPFC correlates positively with VLPFC function in the same cluster, and lower grey matter probability, indicative of atrophy, is associated with increased VMPFC activity. Furthermore, age-related structural changes in the PFC have often been associated with age-related changes in EF, and these areas are also important for adaptive emotional responding, including successful emotion regulation (e.g. Wager *et al.*, 2008). Our results indicate that a relative loss of EF measured outside the scanner is also associated with increased VMPFC activity.

Volumetric studies using tracing of the amygdala suggest structural preservation of the amygdala in healthy aging (Raz *et al.*, 1997; 2004 but see Mather, 2012, for a summary of inconsistent findings). Earlier studies revealed lower amygdala activity in older compared to younger adults in response to fearful faces (Gunning-Dixon *et al.*, 2003; Tessitore *et al.*, 2005) and to complex negative pictures similar to those presented in this study (Mather *et al.*, 2004). However, more recent evidence suggests that older-aged individuals recruit different cortical neural networks when processing these images relative to their younger counterparts (St. Jacques *et al.*, 2010) in the absence of an age effect in amygdala recruitment. While we observed robust amygdala responses to negative images, our results did not reveal any age differences in such responding. The discrepancy in findings across studies point to the importance of providing different age groups with emotional information that is significant for all individuals regardless of age. Finally, activity in left amygdala was positively associated with activity in left VLPFC, as has been often reported in emotion-induction tasks (see e.g. Kober *et al.*, 2008), and this association was independent of age.

In addition, we did not find effects of age on valence categorization of the pictures performed during the scan, nor in dimensional ratings of the pictures provided after the scanning session. That said, it is noteworthy that the valence categorization results suggest that on average 22% of the negative images were judged as either neutral or positive, and 38% of neutral images were categorized as positive or negative. A number of reasons may underlie this finding, including individual differences in evaluating affective information, response bias, and simply button press errors. Thus, this variability and the putative factors underlying it should be considered in future research.

Areas in lateral PFC have long been associated with processes of executive function, specifically in maintaining representations and inhibitory control (e.g. Miller and Cohen, 2001). Age-associated grey matter volume decline in frontal areas has been found to predict executive function (e.g. Zimmerman *et al.*, 2006), and we similarly demonstrate a relationship between grey matter probability in left VLPFC and EF. Recent structural MRI findings demonstrate that various forms of self-control, including motor inhibitory control, voluntary emotion regulation through reappraisal, and drug craving, positively correlate with grey matter integrity in VLPFC specifically (Tabibnia *et al.*, 2011). Using functional MRI, other work suggests a mediating role of the VLPFC in emotion elicitation and regulation (Wager *et al.*, 2008).

Do emotion-relevant processes change with advancing age to a similar extent as executive function? Indeed, positive associations between older adults’ performance on various cognitive functions and the manner in which emotional information is being processed have been reported, including prioritizing positive information and dampening the impact of negative information when cognitive resources are available (see Mather, 2012, for a review). Some research findings, however, suggest that the coupling between cognitive and emotional control may be age-independent (e.g. Winecoff *et al.*, 2011; Opitz *et al.*, 2014). While age was not associated with amygdala responding nor with ratings provided of the pictures, our results indicate an age-related difference in the broader neural architecture supporting emotional processing. The pattern of increased recruitment of VMPFC and reduced VLPFC response to negative pictures with advancing age suggests a decrease in reliance on cognitive control-related processes. This suggestion is supported by the correlations between activity in VMPFC in response to negative images and grey matter probability in left VLPFC, and with measures of executive function. Hence, part of this change may well be due to loss of such lateral PFC structure and function. Alternatively, experience in handling emotional situations built up over a lifetime may be associated with less reliance on executive function-related regulatory processes involving VLPFC, such as active coping, and increasingly relying on more passive, internally motivated coping strategies which could be reflected in increased recruitment of VMPFC.

Research in the cognitive neuroscience of aging (e.g. Davis *et al.*, 2008) has reported a shift of posterior (parietal/occipital) to anterior (frontal) brain activity with advancing age when performing episodic retrieval and visual perception tasks, where activity in lateral PFC correlated with task performance in the older adult group. Davis *et al.* suggest that this age-related increase in PFC activity compensates for a loss of sensory processing reflected in decreased occipital activity. Functional overcompensation—particularly in PFC—and loss of regional specificity (‘dedifferentiation’) in the neural networks underlying specific cognitive functions have also been observed (see review by Reuter-Lorenz and Park, 2010).

What is required in developmental and aging neuroscience, however, is a multimodal assessment of the extent to which age differences in patterns of brain activation are in part or wholly due to structural differences. A few studies incorporated structural imaging data to explain age-related changes in patterns of functional brain imaging, but with mixed results: While some find stronger activity associated with higher grey matter density (within areas in the visual stream, Teipel *et al.*, 2007), others (Kalpouzos *et al.*, 2012) demonstrated higher DLPFC activity accounted for by lower grey matter in that region, and yet others (in an emotional facial expression task, Williams *et al.*, 2006) find no relationship between the BOLD response and grey matter volume in this same region. Finally, a longitudinal study demonstrates decreased frontal function (and structure) in a semantic categorization task (Nyberg *et al.*, 2010). This diversity of findings points to the need to quantify brain structure *and* function, ideally in a longitudinal fashion, to better understand aging-related trajectories in patterns of functional brain activity and associated brain structure integrity.

While the sample size of this study is relatively large compared to similar studies on aging and emotion (e.g. St. Jacques *et al.*, 2010; Dolcos *et al.*, 2014), some of the correlations reported here between brain function and variables collected independently were moderate in magnitude, ranging between |0.2| and |0.3|. Indeed, to identify indirect pathways contributing to changes in brain function across the lifespan, including brain structure, emotion ratings and cognitive performance, larger (and ideally longitudinal) datasets will be required. Further replication of these findings, using cohort data such as CAM-CAN (http://www.cam-can.org), is thus warranted. In addition, the findings reported here were based on a subsample of experimental trials (i.e. trials without face presentations, see Methods) completed by participants to avoid potential confounds of subsequent face presentations in the BOLD response. Note however that reduced power due to averaging over fewer trials may have hampered the detection of more subtle effects.

In conclusion, the findings of this study highlight that, paired with a loss in cognitive function and structural brain changes, aging is associated with decreased lateral PFC recruitment and increased reliance on medial prefrontal cortical networks during picture-induced negative affect processing. Given that emotion reactivity as reflected by amygdala responding is not changed with age per se, we suggest that this functional plasticity serves to maintain adaptive emotional responding. Future research should incorporate multimodal approaches to assess the lifespan development of emotional and cognitive function.

## Footnotes

1 The IAPS numbers are for negative: 1111, 1220, 1275, 1525, 2053, 2205, 2278, 2490, 2700, 2717, 2750, 2799, 2800, 3216, 3280, 5973, 6200, 6210, 6250, 6311, 6562, 6570, 6838, 6840, 7359, 7361, 9000, 9001, 9007, 9008, 9090, 9101, 9140, 9182, 9265, 9280, 9290, 9300, 9301, 9320, 9331, 9373, 9419, 9425, 9426, 9471, 9520, 9561, 9570, 9571, 9584, 9600, 9622, 9630, 9810, 9830, 9902, 9903, 9911, 9925; neutral: 1616, 2038, 2102, 2191, 2200, 2210, 2214, 2215, 2305, 2357, 2385, 2393, 2396, 2397, 2441, 2445, 2480, 2487, 2493, 2499, 2512, 2516, 2579, 2595, 2749, 2840, 2850, 5471, 5520, 5535, 6150, 7006, 7009, 7030, 7036, 7037, 7038, 7041, 7044, 7050, 7100, 7130, 7160, 7161, 7170, 7179, 7180, 7184, 7207, 7235, 7242, 7247, 7249, 7484, 7493, 7500, 7547, 7550, 9070, 9700; positive: 1463, 1540, 1590, 1650, 1660, 1710, 1721, 1731, 1811, 2030, 2040, 2170, 2216, 2222, 2303, 2306, 2345, 2346, 2391, 4610, 4623, 4625, 4626, 5270, 5450, 5470, 5480, 5600, 5660, 5700, 5820, 5849, 7250, 7280, 7289, 7390, 7400, 7430, 7450, 7470, 7502, 7508, 8040, 8090, 8116, 8120, 8162, 8170, 8200, 8220, 8350, 8370, 8371, 8460, 8461, 8496, 8499, 8502, 8531, 8540.

2 Including gender as a control variable did not affect the associations; if any, it made some associations stronger: age negatively correlated with GMP in left VLPFC, *r*(61) = −0.45, *P* < 0.001. Activity in VMPFC remained negatively correlated with GMP in left VLPFC, *r*(61) = −0.24, *P* = 0.031 and average BOLD in VLPFC was correlated with GMP, *r*(61) = 0.31, *P* = 0.008. Associations with EF were also largely unaffected by including gender as a control variable: GMP in left VLPFC, *r*(58)= 0.24, *P* = 0.033; EF and VMPFC activity, *r*(58) = −0.21, *P* = 0.052. Age and EF remained at trend, *r*(58) = −0.17, *P* =0.101.
